# The Nrf2/PGC1*α* Pathway Regulates Antioxidant and Proteasomal Activity to Alter Cisplatin Sensitivity in Ovarian Cancer

**DOI:** 10.1155/2020/4830418

**Published:** 2020-11-26

**Authors:** Xinyue Deng, Nan Lin, Jiaying Fu, Long Xu, Haoge Luo, Yao Jin, Yanan Liu, Liankun Sun, Jing Su

**Affiliations:** Key Laboratory of Pathobiology, Ministry of Education, Department of Pathophysiology, College of Basic Medical Sciences, Jilin University, Changchun, China

## Abstract

Drug resistance remains a barrier in the clinical treatment of ovarian cancer. Proteasomal and antioxidant activities play important roles in tumor drug resistance, and increasing evidence suggests the existence of an interaction between antioxidant and proteasomal activities. However, the mechanism of the synergistic effects of proteasomal activity and antioxidation on tumor drug resistance is not completely clear. In this study, we compared two ovarian cancer cells, A2780 and SKOV3 cells. Among them, SKOV3 cell is a human clear cell carcinoma cell line that is resistant to platinum. We found that compared with the findings in A2780 cells, SKOV3 cells were less sensitive to both proteasomal inhibitor and cisplatin. Proteasomal inhibition enhanced the sensitivity of A2780 cells, but not SKOV3 cells, to cisplatin. Notably, the Nrf2-mediated antioxidant pathway was identified as a resistance mechanism in proteasome inhibitor-resistant cells, but this was not the only factor identified in our research. In SKOV3 cells, PGC1*α* regulated the antioxidant activity of Nrf2 by increasing the phosphorylation of GSK3*β*, and in turn, Nrf2 regulated the transcriptional activity of PGC1*α*. Thus, Nrf2 and PGC1*α* synergistically participate in the regulation of proteasomal activity. Furthermore, the Nrf2/PGC1*α* pathway participated in the regulation of mitochondrial function and homeostasis, further regulating proteasomal activity in SKOV3 cells. Therefore, exploring the roles of PGC1*α* and Nrf2 in the regulation of proteasomal activity by antioxidant and mitochondrial functions may provide new avenues for reversing drug resistance in ovarian cancer.

## 1. Introduction

Ovarian cancer is the most deadly gynecological malignancy, and drug resistance has become a major challenge in its treatment in recent years. Resistance to chemotherapy in tumor cells is related to multiple factors, including the repair of DNA damage, blockade of drug-induced apoptosis, reactive oxygen species- (ROS-) mediated redox state, and protein degradation [1–3]. Current research suggests that exploring the mechanisms of the interaction between different signals can shed light on the mechanisms of tumor drug resistance. Proteasomal activity plays important roles in tumorigenesis and chemotherapy resistance, and abnormally elevated proteasome levels are found in a variety of cancers [4–6]. The proteasome can eliminate oxidative and damaged proteins to reduce oxidative stress damage, and enhanced antioxidant capacity can promote the expression of 20S and 19S proteasome subunits [7, 8]. These findings suggest the existence of an interaction between proteasomal activity and redox function, and exploring this relationship will be more helpful in solving the problem of chemotherapy resistance in ovarian cancer.

The ubiquitin-proteasome system (UPS) maintains cell homeostasis by regulating proteins involved in signal transduction and cell cycle pathways [9]. Inhibition of the proteasome can cause the accumulation of proapoptotic proteins and induce tumor cell apoptosis. However, recent clinical studies found that some tumors are not sensitive to proteasome inhibitors. Proteasome inhibitor-resistant tumors generally have higher expression levels of antioxidant genes [10]. The nuclear factor E2-related factor 2 (Nrf2, gene name NFE2l2)-mediated antioxidant stress pathway was identified as a resistance mechanism in the proteasome inhibitor-resistant phenotype. Nrf2 can regulate the transcriptional activity of proteasome mature protein, which can promote resistance to proteasome inhibitors [11]. This suggests that Nrf2-mediated antioxidant activity may be related to proteasome-mediated tumor resistance.

Nrf2 is an important element of the antioxidant response element (ARE) transcription complex, and it can regulate the expression of various protective genes. Nrf2- and antioxidant-related gene expression is elevated in drug-resistant tumor cells, and its activity is regulated by a variety of factors, including transcription and posttranscription modification. The dissociation of Nrf2 and Kelch-like ECH-associated protein 1 (Keap1) enables Nrf2 to enter the nuclear and regulate the transcription of the oxidase gene [12, 13]. What's more, glycogen synthase kinase 3*β* (GSK3*β*) can inhibit Nrf2 by preventing nuclear accumulation of the CNC-bZIP factor, and evidence has provided that GSK3*β* interferes with Nrf2 transactivating activity and nuclear exclusion of the Nrf2 [14, 15]. Peroxisome proliferator-activated receptor-*γ* coactivator 1*α* (PGC1*α*, gene name PPARGC1A) can also coordinate the expression of multiple antioxidant genes to protect cells against oxidative stress damage. PGC1*α* is involved in the regulation of Nrf2 expression and activity, and studies have confirmed the protein-protein interaction between them [16, 17], but the specific mechanism is not completely clear. Therefore, the mechanism by which PGC1*α* and Nrf2 interact may play a synergistic role in antioxidation, but it is unclear whether their interaction is related to drug resistance.

In addition, the maintenance of mitochondrial redox homeostasis plays an important role in proteasome activity. Mitochondria are the main sources of ROS, and excessive ROS production can increase the burden on the UPS and decrease the proteasomal activity induced by increased oxidation and damaged proteasome subunits [18]. Nrf2 can inhibit mitochondrial ROS production by upregulating heme oxidase (HO-1) and the primary mitochondrial antioxidant enzyme superoxide dismutase 2 (SOD2), thereby inhibiting mitochondrial oxidative stress damage [19, 20]. Mitochondrial protein stability and structural integrity are regulated through interactions with a variety of mitochondrial proteins, including PGC1*α* and PGC1*β* [21–23]. These results suggest that the antioxidation mediated by Nrf2 and PGC1*α* also regulates the homeostasis of mitochondrial function and thus maintains proteasome activity.

In this study, the “UPS-Antioxidation Axis” was taken as the entry point to explore the roles of PGC1*α* and Nrf2 in the regulation of antioxidant and mitochondrial functions in maintaining proteasome activity. The findings provide new ideas for reversing drug resistance in ovarian cancer.

## 2. Methods and Materials

### 2.1. Reagents and Antibodies

The human ovarian cancer cell lines A2780 and SKOV3 were obtained from the Shanghai Cell Bank of Chinese Academy of Sciences (Shanghai, China). Both cell lines were cultured in RPMI-1640 medium (Gibco, Carlsbad, CA, USA). Cisplatin (CDDP) and 3-(4, 5-dimethylthiazol-2-yl)-2,5-diphenyltetrazolium bromide (MTT) were purchased from Sigma-Aldrich (St. Louis, MO, USA). Epoxomicin (Epox), GSK3*β* inhibitor (CHIR99021), and the PGC1*α* activator ZLN005 were purchased from MedChemExpress (Monmouth Junction, NJ, USA). Transfections were performed using Lipofectamine 2000 (Invitrogen, Carlsbad, CA, USA). Anti-Keap1 and anti-lamin B antibodies were obtained from Santa Cruz Biotechnology (Santa Cruz, CA, USA). Anti-p38, anti-GSK3*β*, anti-phospho-p38, anti-phospho-GSK3*β*, and anti-AKT antibodies were acquired from Cell Signaling Technology (Danvers, MA, USA). Anti-Nrf2, anti-Bcl-2, anti-Bax, anti-cleaved caspase 3, and anti-*β*-actin antibodies were procured from Proteintech (Chicago, IL, USA). Total OXPHOS Human WB Antibody Cocktail and anti-PGC1*α* antibodies were purchased from Abcam (Cambridge, MA, USA).

### 2.2. Cell Viability Assay

Cells (10,000 cells/well) were seeded in 96-well plates, incubated overnight, and treated with various reagents for the indicated times. Cells were then incubated with MTT reagent for 4–6 h. Absorbance values were recorded at 570 nm using an enzyme-linked immunosorbent assay reader.

### 2.3. Plasmids and Cell Transfections

The pcDNA3.1 vector (NC) and a full-length human Nrf2 expression vector (pcDNA3.1-Nrf2) were purchased from Genepharma (Shanghai, China). PGC1*α* shRNA and a nontarget sequence (Scr shRNA) were constructed by Genechem (Shanghai, China). The shRNA sequences were as follows: PGC1*α*-shRNA 1, 5′-GTT-ATA-CCT-GTG-ATG-CTT-T-3′; PGC1*α*-shRNA 2, 5′-CAG-CGA-AGA-TGA-AAG-TGA-T-3′; and Scr-shRNA, 5′-TTC-TCC-GAA-CGT-GTC-ACG-T-3′. Cells were transfected using Lipofectamine 2000 according to the manufacturer's instructions.

### 2.4. Luciferase Assay

The *PPARGC1A* promoter region spanning from −2000 to +1 bp was cloned into a pGL4 basic vector. The reporter gene construct and *Renilla* luciferase reporter (pRL-TK) plasmid were transfected into SKOV3 cells alone or together with pcDNA3.1-Nrf2 (Nrf2) or pcDNA3.1 vector (NC) using Lipofectamine 2000. Luciferase activity was determined using a dual-luciferase reporter assay kit (Promega, Madison, WI, USA).

### 2.5. Flow Cytometry

Apoptotic cells were counted using Annexin V-FITC and propidium iodide (PI) (Annexin V Apoptosis Detection Kit, BD Pharmingen, San Jose, CA, USA) according to the manufacturer's protocol. Mitochondrial membrane potential (MMP) and ROS production were determined using JC-1 or DCFH-DA staining (Beyotime Biotechnology, Shanghai, China). Mitotracker™ Red (Sigma-Aldrich) staining was used to evaluate the alteration of mitochondrial mass. The analysis was performed using a BD Accuri C6 flow cytometer (BD Biosciences, New Jersey, USA).

### 2.6. Western Blotting

Whole cells were lysed in RIPA lysis buffer containing protease inhibitors. Lysates diluted with 5x SDS-PAGE loading buffer were boiled at 95°C for 10 min, separated by SDS-PAGE, and then transferred to PVDF membranes. The membranes were blocked with 5% milk followed by incubation with primary antibodies overnight at 4°C. The next day, the membranes were incubated with HRP-conjugated secondary antibodies. The bands were incubated in ECL reagent (Thermo Fisher Scientific, Waltham, MA, USA) for chemiluminescence and visualized using Syngene Bio Imaging (Synoptics, Cambridge, UK).

### 2.7. Immunoprecipitation

Cell lysates were solubilized in NP40 buffer containing a protease inhibitor mixture for 1–2 h at 4°C. After adding monoclonal antibodies, supernatants were rotated at 4°C overnight. The next day, the protein lysates were incubated with 30 *μ*l of protein A/G Plus-agarose beads (Santa Cruz Biotechnology, Inc., Santa Cruz, CA, USA) overnight at 4°C. The agarose beads were washed three times, SDS-PAGE loading buffer was added, and then, samples were analyzed via Western blotting.

### 2.8. Nuclear and Mitochondrial Isolation

Cells were harvested after treatment with various reagents. Nuclear and mitochondrial fractions were extracted using a nuclear fractionation kit and mitochondrial fractionation kit, respectively (Invent Biotechnologies, MN, USA).

### 2.9. ATP Production

Cells were treated with 100 nM Epox for 12 h or transfected with Nrf2 or pcDNA3.1 (vector plasmid was used in the NC group). ATP production was determined using an ATP Bioluminescence Assay Kit (Beyotime Technology). An Omega luminometer was used to measure the values (BMG Labtech, Ortenberg, Germany).

### 2.10. Oxygen Consumption Rates (OCRs)

Cells were seeded in 96-well plates overnight. The next day, cells were incubated with a mixture of reagents and the oxygen-sensitive probe Mito-Xpress (Luxcel Bioscience, Cork, Ireland) and examined using an Omega luminometer for 6 h.

### 2.11. Real-Time Quantitative PCR (RT-qPCR)

Total RNA was extracted using TRIzol reagent (Sigma-Aldrich). The reverse transcription reaction was performed using a SuperScript RT-PCR kit (Promega), and RT-qPCR was performed using the MX3000P instrument (Agilent, USA). mRNA levels were normalized to those of *β*-actin. The primer sequences are *ACTB*: 5′-ATATCGCGTCGCTGGTCGTC-3′ (forward) and 5′-AGGATGGCGTGAGGGAGAGC-3′ (reverse); *PPARGC1A*: 5′-CAGAGAGTATGAGAAGCGAGAG-3′ (forward) and 5′-AGCATCACAGGTATAACGGTAG-3′ (reverse); *NFE2l2*: 5′-ATCAGCAACAGCATGCCCTC-3′ (forward) and 5′-ATGCCACACTGGGACTTGTG-3′ (reverse); *NQO-1*: 5 ′-AGTATCCTGCCGAGTCTGTTCTGG-3′ (forward) and 5′-AATATCACAAGGTCTGCGGCTTCC-3′ (reverse); *GCLC*: 5′-TGTCCGAGTTCAATACAGTTGA-3′ (forward) and 5 ′-ACAGCCTAATCTGGGAAATGAA-3′ (reverse); *G6PDH*: 5′-TCACCAAGAACATTCACGAGTC-3′ (forward) and 5′-GAAGATCCTGTTGGCAAATCTC-3′ (reverse); *HMOX-1*: 5′-CCTCCCTGTACCACATCTATGT-3′ (forward) and 5′-GCTCTTCTGGGAAGTAGACAG-3′ (reverse); and *SOD2*: 5′-AACCTCACATCAACGCGCAGATC-3′ (forward) and 5′-CTCCTGGTACTTCTCCTCGGTGAC-3′ (reverse).

### 2.12. Determination of the Relative mtDNA Copy Number

Total DNA was extracted using a TIANamp Genomic DNA kit (Tiangen Biotech Co., Ltd., Beijing, China). qPCR was used to measure the mitochondrial-encoded nicotinamide adenine dinucleotide dehydrogenase 1 (ND1) level relative to that of nuclear-encoded gene 18S rRNA (18S), as described previously [24]. Each sample was analyzed in triplicate using the MX3000P instrument. The relative mtDNA copy number was calculated as the ratio of ND1 to 18S and was normalized to the respective control group. The primer sequences are *18*s *rRNA*: 5′-TAGAGGGACAAGTGGCGTTC-3′ (forward) and 5′-CGCTGAGCCAGTCAGTGT-3′ (reverse) and *ND1*: 5′-CACCCAAGAACAGGGTTTGT-3′ (forward) and 5′-TGGCCATGGATTGTTGTTAA-3′ (reverse).

### 2.13. Immunofluorescence Staining and Microscopy

Cells were washed with PBS, fixed with 4% paraformaldehyde for 20 min, and permeabilized with 0.1% Triton X-100 for 8 min. After blocking with 5% bovine serum albumin for 30 min, cells were incubated with primary antibody overnight at 4°C. After PBS washing, the cells were incubated at room temperature for 1 h in the dark with FITC/Texas Red-conjugated secondary antibodies (Proteintech). The images were observed on an Echo-lab Revolve microscope (CA, USA).

### 2.14. Statistical Analysis

Data are expressed as the mean ± SD. ^∗^*P* < 0.05 was considered statistically significant. Statistical analysis was performed with GraphPad Prism 5 (La Jolla, CA, USA). All experiments were repeated at least three times.

## 3. Results

### 3.1. Proteasome Inhibition Can Enhance the Sensitivity of A2780 Cells to Cisplatin, but Not SKOV3 Cells

**Figure 1:**
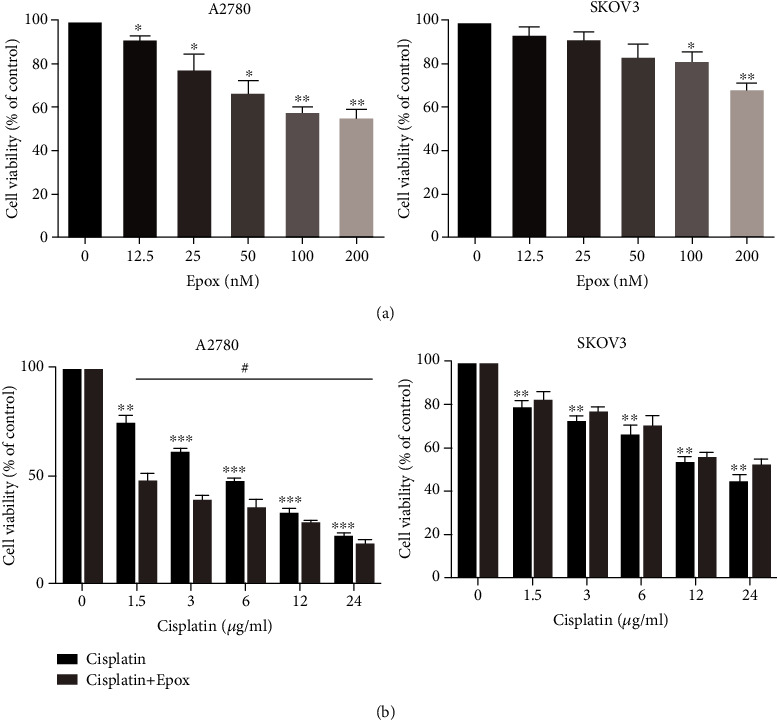
Proteasome inhibition can enhance the sensitivity of A2780 cells to cisplatin compared with SKOV3 cells. (a) A2780 and SKOV3 cells were treated with different concentrations of Epox for 24 h, and cell viability was determined using the MTT assay. Data are presented as the mean ± SD, *n* = 3, ^∗^*P* < 0.05, ^∗∗^*P* < 0.01 compared with the respective controls. (b) A2780 and SKOV3 cells were treated with 100 nM Epox and different concentrations of cisplatin for 24 h, and cell viability was determined using the MTT assay. Data are presented as the mean ± SD (*n* = 3), ^∗∗^*P* < 0.01, ^∗∗∗^*P* < 0.001 compared with the respective control, ^#^*P* < 0.05 compared with the cisplatin-treated groups.

Drug resistance remains an important obstacle that limits the efficacy of platinum-based treatment in ovarian cancer [25], and targeting the ubiquitin-proteasome pathway can overcome cancer chemoresistance [26]. The MTT assay illustrated that compared with the findings in SKOV3 cells, the proteasome inhibitor Epox (a new-generation proteasome inhibitor that can noncovalently bind to the subunits of the proteasome in an irreversible manner [27]) decreased the viability of A2780 cells in a concentration-dependent manner (Figure 1(a)). As shown in Figure 1(b), the IC_50_ values of cisplatin in A2780 and SKOV3 cells were 5.5 ± 0.18 and 17.89 ± 4.02 *μ*g/ml, respectively. This demonstrated that SKOV3 cells were less sensitive to both cisplatin and proteasome inhibition. To further determine the effect of proteasomes on the sensitivity of ovarian cancer cells to cisplatin, we treated A2780 and SKOV3 cells with 100 nM Epox and different concentrations of cisplatin for 24 h and detected survival using MTT assays. The results demonstrated that Epox increased the sensitivity of A2780 cells, but not SKOV3 cells, to cisplatin. These results suggested that the proteasome plays an important role in cisplatin resistance in SKOV3 cells.

### 3.2. Proteasome Inhibition Promotes Nrf2 Localization to the Nucleus through the Keap1/Nrf2 Pathway

**Figure 2:**
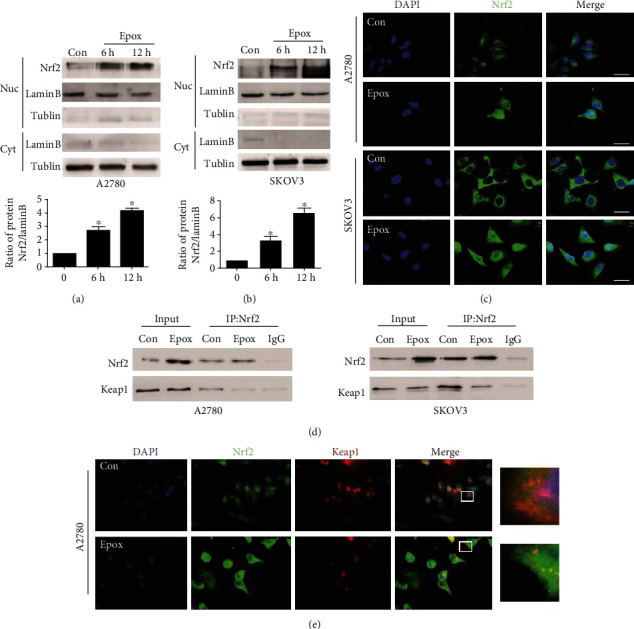
Proteasome inhibition promotes nuclear factor E2-related factor 2 (Nrf2) localization to the nucleus through the Kelch-like ECH-associated protein 1 (Keap1)/Nrf2 pathway. (a, b) The nuclear proteins of A2780 and SKOV3 cells were collected after treatment with 100 nM Epox for 6 or 12 h, and Nrf2 levels were measured via Western blotting. Data are presented as the mean ± SD, *n* = 3, ^∗^*P* < 0.05 compared with the respective controls. A2780 and SKOV3 cells were treated with 100 nM Epox for 12 h. (c) Immunofluorescence staining was used to determine the location of Nrf2 in the nucleus (magnification, ×400). (d) Immunoprecipitation was performed using anti-Nrf2 antibody followed by Western blotting using anti-Nrf2 and anti-Keap1 antibodies. (e) Immunofluorescence staining was used to assess the colocalization of Nrf2 with Keap1 (magnification, ×400).

Although inhibition of the proteasome pathway has emerged as an important strategy for anticancer therapy [28], not all cancer cells are sensitive to proteasome inhibitors. Studies have identified the Nrf2 pathway as one of the mechanisms of resistance to proteasome inhibitors [29]. To investigate whether Nrf2 is involved in resistance to proteasome inhibitors, the expression of Nrf2 in the nucleus was examined. After treatment with Epox for 12 h, an increased expression of Nrf2 in the nucleus was observed in both A2780 and SKOV3 cells (Figures 2(a) and 2(b)). The immunofluorescence assay also revealed that the localization of Nrf2 in the nucleus was enhanced by Epox treatment (Figure 2(c)). As the Keap1/Nrf2 pathway plays an important role in the stabilization of Nrf2, we further detected the colocalization of Keap1 and Nrf2. The immunoprecipitation and immunofluorescence results revealed that Keap1 and Nrf2 colocalization was decreased by Epox treatment (Figures 2(d) and 2(e)). These results suggested that proteasome inhibition promotes Nrf2 localization to the nucleus through the Keap1/Nrf2 pathway.

### 3.3. Proteasome Inhibition Enhances Antioxidant and Antiapoptosis Activity in SKOV3 Cells Compared with A2780 Cells

**Figure 3:**
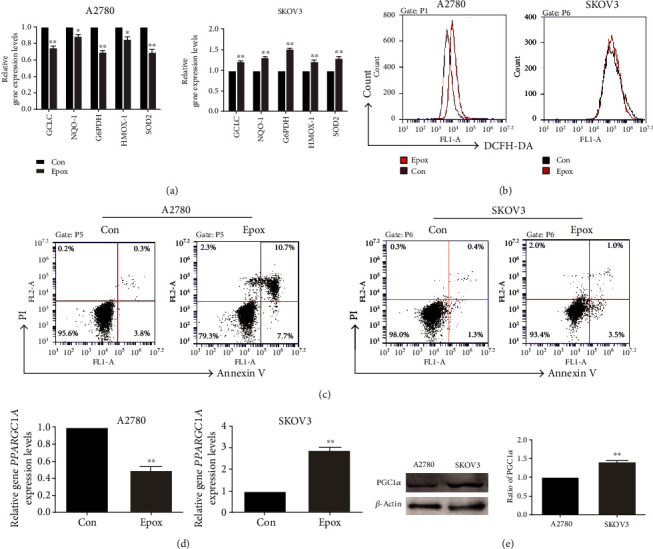
Proteasome inhibition enhances the antioxidant and antiapoptosis activity in SKOV3 cells but not A2780 cells. A2780 and SKOV3 cells were both treated with 100 nM Epox for 12 h. (a) RT-qPCR was used to detect the mRNA expression of *GCLC*, *NQO-1*, *SOD2*, *HMOX-1*, and *G6PDH*. Data are presented as the mean ± SD, *n* = 3, ^∗^*P* < 0.05, ^∗∗^*P* < 0.01 compared with the respective controls. (b) DCFH-DA staining was used to evaluate reactive oxygen species (ROS) levels. (c) Annexin V/PI staining was used to evaluate apoptosis levels after exposure to Epox for 24 h. (d) Relative *PPARGC1A* mRNA expression was measured by RT-qPCR after exposure to Epox for 12 h. Data are presented as the mean ± SD, *n* = 3, ^∗∗^*P* < 0.01 compared with the respective controls. (e) Western blot analysis of peroxisome proliferator-activated receptor-*γ* coactivator 1*α* (PGC1*α*) expression in A2780 and SKOV3 cells. Data are presented as the mean ± SD, *n* = 3, ^∗∗^*P* < 0.01 compared with A2780 cells.

Nrf2 translocates to the nucleus to activate the transcription of ARE-containing genes [30], and thus, we determined the expression of genes in the Nrf2 pathway. To our surprise, the expression of NQO-1, GCLC, HO-1, SOD2, and G6PDH genes was all reduced after treatment with Epox for 12 h in A2780 cells, whereas the opposite results were obtained in SKOV3 cells (Figure 3(a)). To further determine the effect of proteasome inhibition on redox levels, we detected ROS levels via flow cytometry. The results revealed that Epox increased ROS levels in A2780 cells, whereas no significant changes were noted in SKOV3 cells (Figure 3(b)). In addition, Annexin V/PI staining revealed that the rate of apoptosis after exposure to Epox for 24 h was higher in A2780 cells than in SKOV3 cells (Figure 3(c)). This suggested that the increased expression of Nrf2 in the nucleus did not necessarily exert antioxidant activity after proteasome inhibition; there may be regulatory factors regulating its transcriptional activity. It has been reported that PGC1*α* is also associated with the expression of mitochondrial ROS-detoxifying enzymes [31], and the activity of Nrf2 dose depends on PGC1*α* upon redox imbalance [17]. Therefore, we examined the gene expression of PGC1*α* and found that Epox can reduce its level in A2780 cells but increase its level in SKOV3 cells (Figure 3(d)). Notably, the expression of PGC1*α* was higher in SKOV3 cells than in A2780 cells (Figure 3(e)). These results indicated that compared with A2780 cells, proteasome inhibition enhances antioxidant and antiapoptosis activity in SKOV3 cells, and this may have resulted from the synergistic roles of Nrf2 and PGC1*α*.

### 3.4. PGC1*α* Regulates the Antioxidant Activity of Nrf2 through GSK3*β* after Proteasome Inhibition in SKOV3 Cells

**Figure 4:**
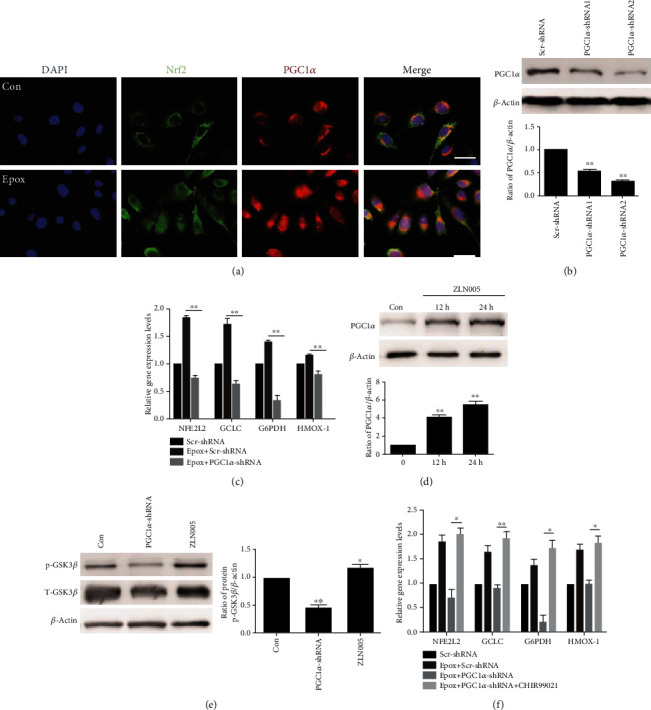
Proliferator-activated receptor-*γ* coactivator 1*α* (PGC1*α*) regulates the antioxidant activity of Nrf2 through glycogen synthase kinase 3*β* (GSK3*β*) after proteasome inhibition in SKOV3 cells. (a) SKOV3 cells were treated with Epox for 12 h. The colocalization of Nrf2 and PGC1*α* in the nucleus was determined via staining and observed via fluorescence microscopy (magnification, ×400). Western blotting was used to measure the expression of PGC1*α* after transfection with Scr-shRNA or PGC1*α*-shRNA plasmids (b) or treatment with 20 *μ*M ZLN005 for 12 or 24 h (d). Data are presented as the mean ± SD, *n* = 3, ^∗∗^*P* < 0.01 compared with the respective controls. (c) After transfection with Scr-shRNA or PGC1*α*-shRNA plasmids, SKOV3 cells were treated with 100 nM Epox for 12 h. RT-qPCR was used to detect the expression of *NFE2l2*, *GCLC*, *HMOX-1*, and *G6PDH* mRNA. Data are presented as the mean ± SD, *n* = 3, ^∗∗^*P* < 0.01. (e) SKOV3 cells were treated with 20 *μ*M ZLN005 for 24 h or transfected with PGC1*α* shRNA plasmids. Western blot analysis of the expression of p-GSK3*β* and GSK3*β*. Data are presented as the mean ± SD, *n* = 3, ^∗^*P* < 0.05, ^∗∗^*P* < 0.01 compared with the control. (f) After transfection with Scr-shRNA or PGC1*α*-shRNA plasmids, cells were treated with Epox (100 nM, 12 h) with or without CHIR99021 (5 *μ*M, 36 h). Relative *NFE2l2*, *GCLC*, *HMOX-1*, and *G6PDH* mRNA expression was measured by RT-qPCR. Data are presented as the mean ± SD, *n* = 3, ^∗^*P* < 0.05, ^∗∗^*P* < 0.01.

There is protein-protein interaction between PGC1*α* and Nrf2. In our experiment, proteasome inhibition increased the colocalization of Nrf2 and PGC1*α* in the nucleus (Figure 4(a)). PGC1*α* could coactivate Nrf2 and assist the induction of antioxidant genes. So, we investigated whether PGC1*α* was involved in the regulation of Nrf2 antioxidant activity after proteasome inhibition in SKOV3 cells. RT-qPCR analyzed the expression of genes in the Nrf2 pathway, and the result illustrated that the increased levels of Nrf2 and antioxidant genes after Epox treatment were reversed by PGC1*α* silencing (Figures 4(b) and 4(c)). GSK3*β* interferes with Nrf2 transactivating activity, so we further investigated whether PGC1*α* regulated Nrf2 activity through GSK3*β*. Results from our Western blot analysis demonstrated that PGC1*α* silencing reduced the level of Ser9 phosphorylated GSK3*β*, whereas ZLN005 (a PGC1*α* transcription activator) enhanced its level (Figures 4(d) and 4(e)). When combined with GSK3*β* inhibitor (CHIR99021) in the presence of PGC1*α* silencing, the decreased levels of Nrf2 and antioxidant genes were reversed (Figure 4(f)). These results indicated that in SKOV3 cells, PGC1*α* regulates Nrf2 activity via GSK3*β* inactivation after proteasome inhibition. But whether GSK3*β* regulates Nrf2 directly or indirectly after Epox treatment and the specific mechanism of regulation needs to be further explored in future research.

### 3.5. Nrf2 Regulates PGC1*α* at the Transcriptional Level in SKOV3 Cells

**Figure 5:**
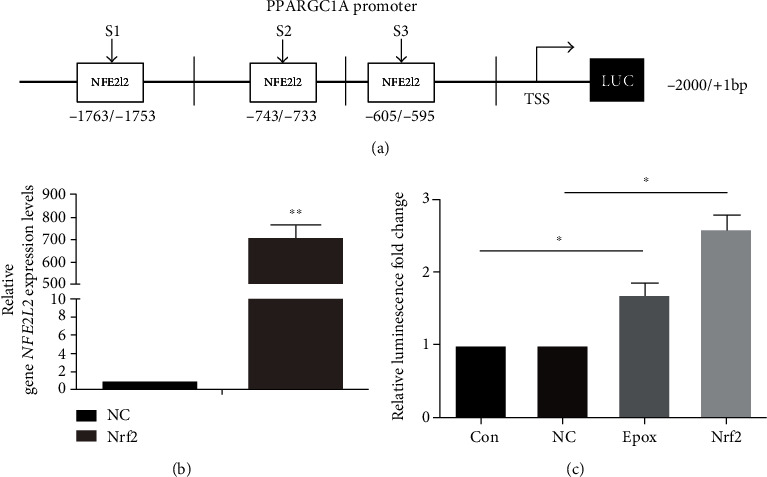
Nuclear factor E2-related factor 2 (Nrf2) regulates peroxisome proliferator-activated receptor-*γ* coactivator 1*α* (PGC1*α*) at the transcriptional level in SKOV3 cells. (a) Schematic diagram of the sequence spanning from −2000 to +1 relative to the translational start site (TSS) of the PGC1*α* promoter containing the Nrf2 motif. (b) SKOV3 cells were transfected with Nrf2 or pcDNA3.1 plasmids (vector plasmid as the NC group). Relative *NFE2l2* mRNA expression was measured by RT-qPCR. Data are presented as the mean ± SD, *n* = 3, ^∗∗^*P* < 0.01 compared with NC group. (c) The luciferase activities of Epox-treated or Nrf2 overexpression SKOV3 cells transfected with pGL4-PGC1*α* and *Renilla* luciferase reporter (pRL-TK) plasmids were determined using dual-luciferase reporter assays. Data are presented as the mean ± SD, *n* = 3, ^∗^*P* < 0.05.

The PGC1*α* promoter contains a conserved ARE sequence that can be bound by Nrf2, so we also determined whether Nrf2 directly binds to the PGC1*α* promoter after proteasome inhibition in SKOV3 cells. We analyzed the PGC1*α* promoter sequence using the JASPAR database (http://jaspardev.genereg.net/) and predicted three potential Nrf2-binding fragments (Figure 5(a)). To further confirm that Nrf2 regulates PGC1*α* promoter activity, we performed a dual-luciferase reporter assay. The PGC1*α* promoter fragment −2000/+1 bp was cloned in to the pGL4-basic vector and then cotransfected with a *Renilla* luciferase reporter plasmid into SKOV3 cells. We observed that both overexpression of Nrf2 or treatment with Epox markedly upregulated the transcriptional activity of PGC1*α* (Figures 5(b) and 5(c)). These data provide evidence that Nrf2 regulates PGC1*α* expression at the transcriptional level in SKOV3 cells.

### 3.6. Nrf2 Activation Enhances Mitochondrial Function in SKOV3 Cells

**Figure 6:**
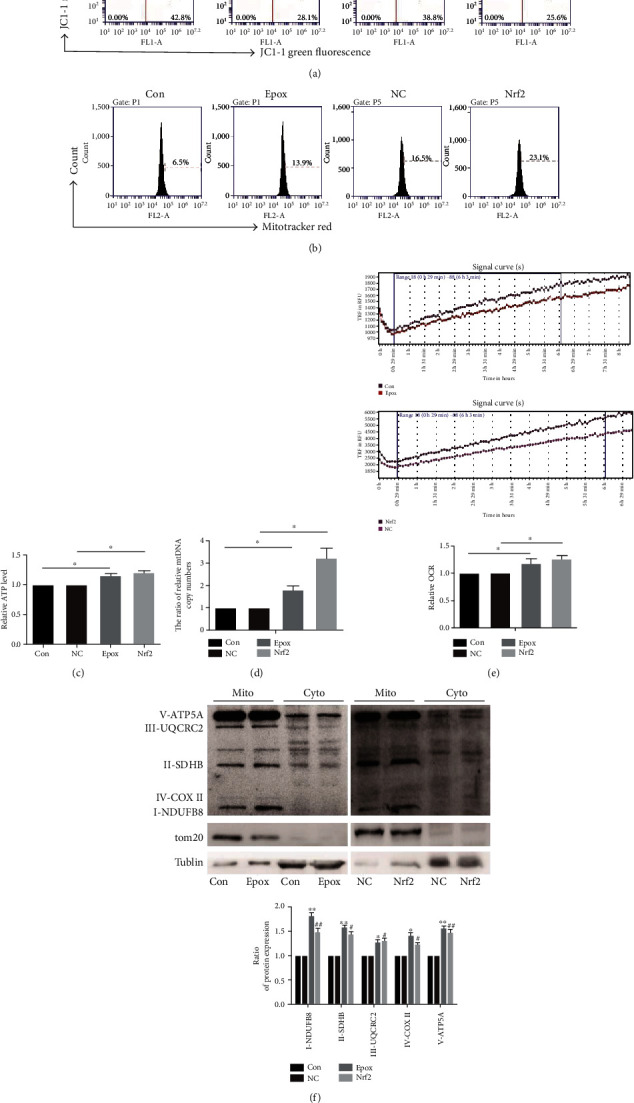
Nuclear factor E2-related factor 2 (Nrf2) activation enhances mitochondrial function in SKOV3 cells. SKOV3 cells were treated with 100 nM Epox for 12 h or transfected with Nrf2 or pcDNA3.1 plasmids (vector plasmid as the NC group). (a) JC-1 staining was used to evaluate MMP, and (b) MitoTracker™ Red staining was used to evaluate the alteration of mitochondrial mass via flow cytometry. (c) ATP production was determined using an ATP Bioluminescence Assay Kit, and (d) relative mtDNA copy numbers were determined by RT-qPCR. Data are presented as the mean ± SD, *n* = 3, ^∗^*P* < 0.05. (e) The oxygen consumption rate was detected using fluorescent oxygen-sensitive probes. Data are presented as the mean ± SD, *n* = 3, ^∗^*P* < 0.05. (f) Mitochondrial proteins were collected, and the expression of mitochondrial respiratory chain proteins was analyzed via Western blotting. Data are presented as the mean ± SD, *n* = 3, ^∗^*P* < 0.05, ^∗∗^*P* < 0.01 compared with the control, ^#^*P* < 0.05, ^##^*P* < 0.01 compared with NC.

In addition to antioxidant properties, Nrf2 has an emerging role in mitochondrial function, and this may be through activating the transcription of PGC1*α*. To confirm this, we treated SKOV3 cells with Epox for 12 h or overexpressed Nrf2. JC-1 fluorescent staining was used to measure mitochondrial membrane potential (MMP, ∆*Ψ*m) (Figure 6(a)), and MitoTracker Red staining was used to measure mitochondrial mass (Figure 6(b)). The results indicated that Epox treatment or Nrf2 overexpression increased MMP and mitochondrial mass. ATP levels and mitochondrial DNA (mtDNA) copy numbers were both enhanced after treatment with Epox or Nrf2 overexpression (Figures 6(c) and 6(d)). Furthermore, we analyzed the effect of Nrf2 on the oxygen consumption rates (OCR) and found that exposure to Epox or overexpression of Nrf2 increased the OCR in SKOV3 cells (Figure 6(e)). To detect the effect of Nrf2 activation on the mitochondrial chain, we extracted mitochondria and used Western blotting to measure protein expression. As shown in Figure 6(f), Epox treatment or Nrf2 overexpression significantly increased the expression of complex I (NDUFB8), complex II (SDHB), complex III (UQCRC2), complex IV (COXII), and complex V (ATP5A). However, mitochondrial-related functions were downregulated in A2780 cells (Supplementary Figure 1), further indicating that the increased nucleus expression of Nrf2 after treatment with Epox did not have activity in A2780 cells. In summary, we conclude that the activation of Nrf2 through Epox treatment or Nrf2 overexpression enhanced mitochondrial function in SKOV3 cells.

### 3.7. PGC1*α* Silencing Combined with Epox Treatment Promotes Apoptosis by Reducing Mitochondrial Function

**Figure 7:**
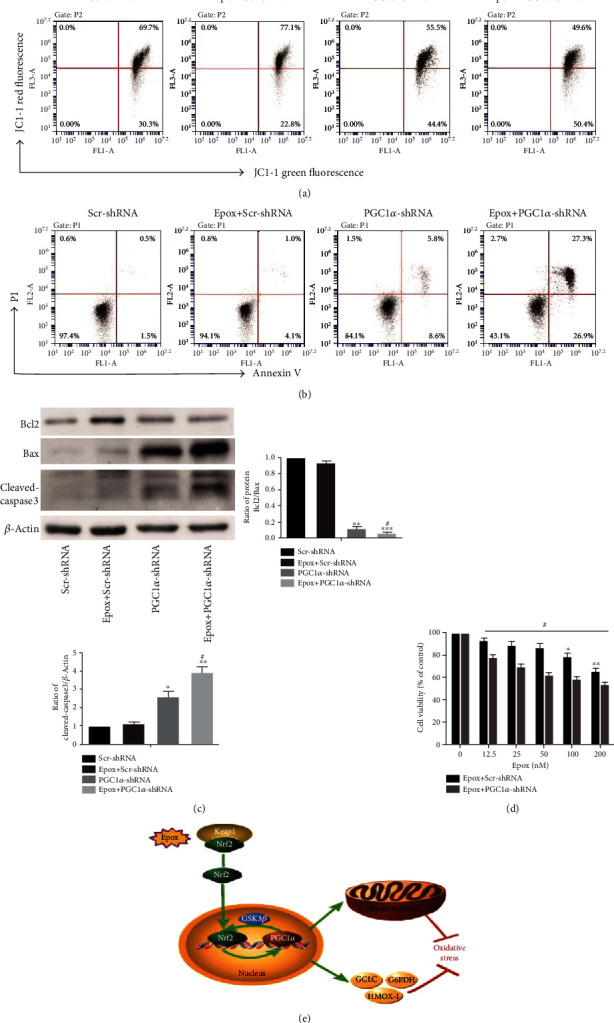
Proliferator-activated receptor-*γ* coactivator 1*α* (PGC1*α*) silencing combined with Epox treatment promotes apoptosis by reducing mitochondrial function. (a) SKOV3 cells were transfected with Scr-shRNA or PGC1*α*-shRNA plasmids for 24 h and/or treated with 100 nM Epox for 12 h. JC-1 staining was used to evaluate MMP. SKOV3 cells were transfected with Scr-shRNA or PGC1*α*-shRNA plasmids for 24 h and/or treated with 100 nM Epox for 24 h. (b) Annexin V/PI staining was used to evaluate the apoptotic fraction, and (c) apoptosis protein expression was analyzed via Western blotting. Data are presented as the mean ± SD, *n* = 3, ^∗^*P* < 0.05, ^∗∗^*P* < 0.01, ^∗∗∗^*P* < 0.001 compared with the control, ^#^*P* < 0.05 compared with treatment with Epox+Scr-shRNA and PGC1*α*-shRNA. (d) SKOV3 cells were transfected with Scr-shRNA or PGC1*α*-shRNA plasmids and/or treated with 100 nM Epox for 24 h, and cell viability was determined using the MTT assay. Data are presented as the mean ± SD, *n* = 3, ^∗^*P* < 0.05, ^∗∗^*P* < 0.01 compared with the control, ^#^*P* < 0.05 compared with Epox+Scr-shRNA. (e) Proposed model of the antioxidant activity regulated by Nrf2 and PGC1*α* after Epox treatment.

To further verify whether Nrf2/PGC1*α* signaling-mediated antioxidation and mitochondrial function play important roles in Epox resistance in SKOV3 cells, PGC1*α* was silenced via transient shRNA transfection. Decreased MMP is an early event of apoptosis. Flow cytometry was used to determine the effect of PGC1*α* silencing on MMP. The results demonstrated that PGC1*α* silencing reduced MMP, and the effect was further exacerbated by combined treatment with Epox (Figure 7(a)). Furthermore, Annexin V/PI staining revealed that PGC1*α* silencing increased the rate of apoptosis, which was further increased in cells treated with PGC1*α* shRNA and Epox (Figure 7(b)). In addition, we examined the expression of Bcl2 family members in SKOV3 cells after treatment with Epox and transfection with PGC1*α* shRNA. The Bcl2/Bax ratio was reduced by PGC1*α* silencing, and further decreases were observed in cells treated with both Epox and PGC1*α* shRNA. The expression of cleaved caspase 3 was also intensified in the combination treatment group (Figure 7(c)). What is more, the MTT assay demonstrated that PGC1*α* silencing increased the sensitivity of SKOV3 cells to Epox compared with the Scr-shRNA group (Figure 7(d)). These results suggested that PGC1*α* silencing combined with Epox treatment promotes apoptosis by reducing mitochondrial function.

## 4. Discussion

Chemotherapy resistance remains a barrier in the clinical treatment of ovarian cancer. When considering chemotherapy resistance, different aspects of drug resistance must be considered comprehensively [32]. Protein degradation and antioxidation are considered two factors of resistance to chemotherapy. Abnormally increased proteasomal activity has been observed in a variety of cancers, including breast cancer, colon cancer, and leukemia [6, 7, 33]. Kwak et al. demonstrated that antioxidants can induce the expression of multiple proteasome subunits in mouse fibroblasts [8]. Therefore, antioxidants are involved in the regulation of proteasomal activity, and they play important roles in tumorigenesis and development. In this study, we compared two ovarian cancer cells, A2780 and SKOV3. SKOV3 cell was chosen because it is platinum-resistant and possesses several key oncogenic characteristics, like epidermal growth factor receptor (EGFR) overexpression and p53 mutation [34, 35]. In our study, compared with the findings in A2780 cells, SKOV3 cells were less sensitive to both proteasome inhibitors and cisplatin. When the proteasome was inhibited, the antioxidant capacity was decreased, and the sensitivity of A2780 cells to cisplatin was increased. The opposite results were observed in SKOV3 cells. This suggests that proteasomes and antioxidants may be involved in the differing sensitivity of A2780 and SKOV3 cells to cisplatin.

Proteasomes are closely involved in the regulation of multiple pathways within cells, and increased proteasomal activity is essential for tumor cell survival based on their ability to regulate tumor cell drug resistance through multiple pathways [7, 36, 37]. Inhibition of the proteasome can further increase oxidative stress in tumor cells, thereby promoting tumor cell apoptosis. However, recent studies have found that not all tumor cells are sensitive to proteasome inhibitors. A study in multiple myeloma demonstrated that the Nrf2 signaling pathway may be involved in proteasome-mediated tumor resistance [38]. Nrf2 has seven Neh domains, and of these, Neh1 can combine with Maf protein to form a heterodimer and bind to AREs and regulate the expression of antioxidant genes such as HO-1 and NQO-1 after Nrf2 translocates to the nucleus [39, 40]. In our study, although proteasome inhibition increased the nuclear expression of Nrf2 in both A2780 and SKOV3 cells, inhibiting the proteasome significantly reduced antioxidant levels and enhanced ROS production in A2780 cells. Conversely, the opposite results were obtained in SKOV3 cells. This suggested that the increased nuclear expression of Nrf2 did not necessarily exert antioxidant activity after proteasome inhibition; there may be regulatory factors regulating its transcriptional activity. Notably, PGC1*α* can regulate the expression of various antioxidant genes and play an important regulatory role in redox homeostasis [41]. Bruns et al. reported that PGC1*α* plays an important role in the clearance of ROS by regulating SOD1 and SOD2 in GBM glioblastoma cells [42]. In addition, a study using an aging disease model found that PGC1*α* can regulate Nrf2 expression and play a synergistic role in antioxidation [17]. Our study compared the expression of PGC1*α* in two cell lines and found that PGC1*α* expression was significantly higher in SKOV3 cells than in A2780 cells. After inhibiting proteasomes, PPARGC1A gene expression was increased in SKOV3 cells but decreased in A2780 cells, suggesting that PGC1*α* is also involved in proteasome inhibition-mediated antioxidant regulation. Thus, PGC1*α* and Nrf2 may jointly mediate the antioxidant regulation of proteasome inhibitor resistance in SKOV3 cells.

Recent studies revealed that PGC1*α* coactivate Nrf2, and there is a protein-protein interaction between PGC1*α* and Nrf2. Here, we deeply investigated the mutual regulation between PGC1*α* and Nrf2 in SKOV3 cells. First, we found that proteasome inhibition in SKOV3 cells can increase the colocalization of Nrf2 and PGC1*α* in the nucleus. Cherry et al. demonstrated that PGC1*α* was important for the activation of gene downstream of Nrf2 during sepsis [43]. In our study, the increased Nrf2 and antioxidant gene mRNA levels after Epox treatment were reversed by PGC1*α* silencing, which suggested that PGC1*α* was involved in the antioxidant regulation by Nrf2 during proteasome inhibition. Furthermore, Nrf2 activity is mediated by GSK3*β*-TrCP-dependent Cul1-based ubiquitin ligase. Choi et al. demonstrated that PGC1*α* can upregulate Nrf2 through the p38/GSK3*β* pathway to protect HK-2 cells against hydrogen peroxide-induced oxidative stress injury [44]. This is consistent with our findings that PGC1*α* can mediate Nrf2 activity through the inactivation of GSK3*β* in SKOV3 cells. Therefore, PGC1*α* can regulate the antioxidant activity of Nrf2 via GSK3*β* after proteasome inhibition. But whether GSK3*β* regulates Nrf2 directly or indirectly after Epox treatment and the specific mechanism of regulation needs to be further explored in future research.

Although Clark and Simon previously proposed that the PGC1*α* promoter region contains a conserved ARE sequence that can be bound by Nrf2, it is unclear whether a transcription factor and PGC1*α* can form a complex to regulate Nrf2 expression or whether Nrf2 can directly bind to the PGC1*α* promoter region [41, 45]. After analyzing the PGC1*α* promoter sequence, three potential Nrf2-binding fragments were found. The dual-luciferase reporter assay found that Nrf2 upregulated the transcriptional activity of PGC1*α* after proteasome inhibition. The aforementioned data provided evidence that Nrf2 regulates PGC1*α* expression at the transcriptional level in SKOV3 cells. However, the specific molecular mechanism and promoter elements responsible for Nrf2 regulation need to be further explored. So far, we have revealed a feedback loop between PGC1*α* and Nrf2, which enables them to play synergistic roles in the antioxidant function that maintains proteasomal activity.

Ross proposed that efficient UPS activity is essential for maintaining mitochondrial health, which is also necessary for maintaining UPS efficiency [18]. Inhibition of OXPHOS in mouse cortical neurons can inhibit proteasomal activity and protein ubiquitin [46]. Inhibition of ATP production via complex I inhibition can also inhibit proteasomal activity in primary mesencephalic cells [47]. Interestingly, Nrf2 also plays an inestimable role in regulating mitochondrial function. Nrf2 can regulate mitochondrial function through interactions with various mitochondrial proteins such as PGC1*α*. In SKOV3 cells, proteasome inhibition can enhance MMP, mitochondrial number, and mitochondrial complex protein expression, which in turn increase ATP and oxygen consumption. Overexpression of Nrf2 produced the same results, further proving that inhibiting proteasomes can enhance mitochondrial function through Nrf2. This is consistent with the findings of Shen et al., who reported that Nrf2 agonists can induce related mitochondrial functions such as mitochondrial proliferation in mouse 3T3-L1 adipocytes [48]. In addition, mitochondrial redox homeostasis is also important for maintaining proteasome activity. Gao et al. revealed that Nrf2 can upregulate mitochondrial antioxidant enzymes such as Sirt3 and SOD2 to maintain mitochondrial ROS homeostasis to protect neurons from oxidative stress [20]. In SKOV3 cells, we also observed that increased nuclear Nrf2 expression can upregulate the gene expression of SOD2. This indicated that the Nrf2/PGC1*α* pathway also participated in the regulation of proteasomal activity through the regulation of mitochondrial function and homeostasis in SKOV3 cells.

Finally, to further verify that the Nrf2/PGC1*α* pathway plays a role in resistance to proteasome inhibitors, we transfected shRNA-PGC1*α* plasmids into SKOV3 cells. The results illustrated that silencing of PGC1*α* could significantly promote apoptosis and decrease mitochondrial membrane potential of SKOV3 cells. When combined with Epox, apoptosis activity was further increased, and mitochondrial membrane potential was further decreased. This demonstrated that the Nrf2/PGC1*α* pathway is involved in the resistance of SKOV3 cells to proteasome inhibitors.

In summary, Nrf2 cooperates with PGC1*α* to mediate antioxidant function and mitochondrial function, thereby regulating the maintenance of proteasome activity and influencing differences in cisplatin sensitivity in ovarian cancer cells (Figure 7(e)).

## Data Availability

The datasets in the current study are available from the corresponding authors on reasonable request.

## Abbreviations

PGC1*α*: Peroxisome proliferator-activated receptor coactivator
MTT: 3-(4,5-Dimethylthiazol-2-yl)-2,5-diphenyltetrazolium bromide
GSK3*β*: Glycogen synthase kinase
Nrf2: Nuclear factor (nuclear factor E2-related factor 2)
ND1: Nicotinamide adenine dinucleotide dehydrogenase 1
UPS: Ubiquitin-proteasome system.

## Supplementary Materials

Supplementary Materials EPOX decreases mitochobdrial function in A2780 cells.
