# Supplementary material for: The Nrf2/PGC1α Pathway Regulates Antioxidant and Proteasomal Activity to Alter Cisplatin Sensitivity in Ovarian Cancer
Source: Oxid Med Cell Longev. 2020 Nov 26;2020:4830418. doi: 10.1155/2020/4830418 (PMC7714579; doi:10.1155/2020/4830418)

**Supplementary Figure 1**

**Epox decreases mitochobdrial function in A2780 cells.** A2780 cells were treated with 100 nM Epox for 12 h (A) JC-1 staining was used to evaluate MMP, and (B) MitoTracker™ Red staining was used to evaluate the alteration of mitochondrial mass via flow cytometry. (C) ATP production was determined using an ATP Bioluminescence Assay Kit, and (D) relative mtDNA copy numbers were determined by RT-qPCR. Data are presented as the mean ± SD, n = 3, **P* < 0.05 ***P*<0.01. (E) Mitochondrial proteins were collected, and the expression of mitochondrial respiratory chain proteins was analyzed via western blotting.


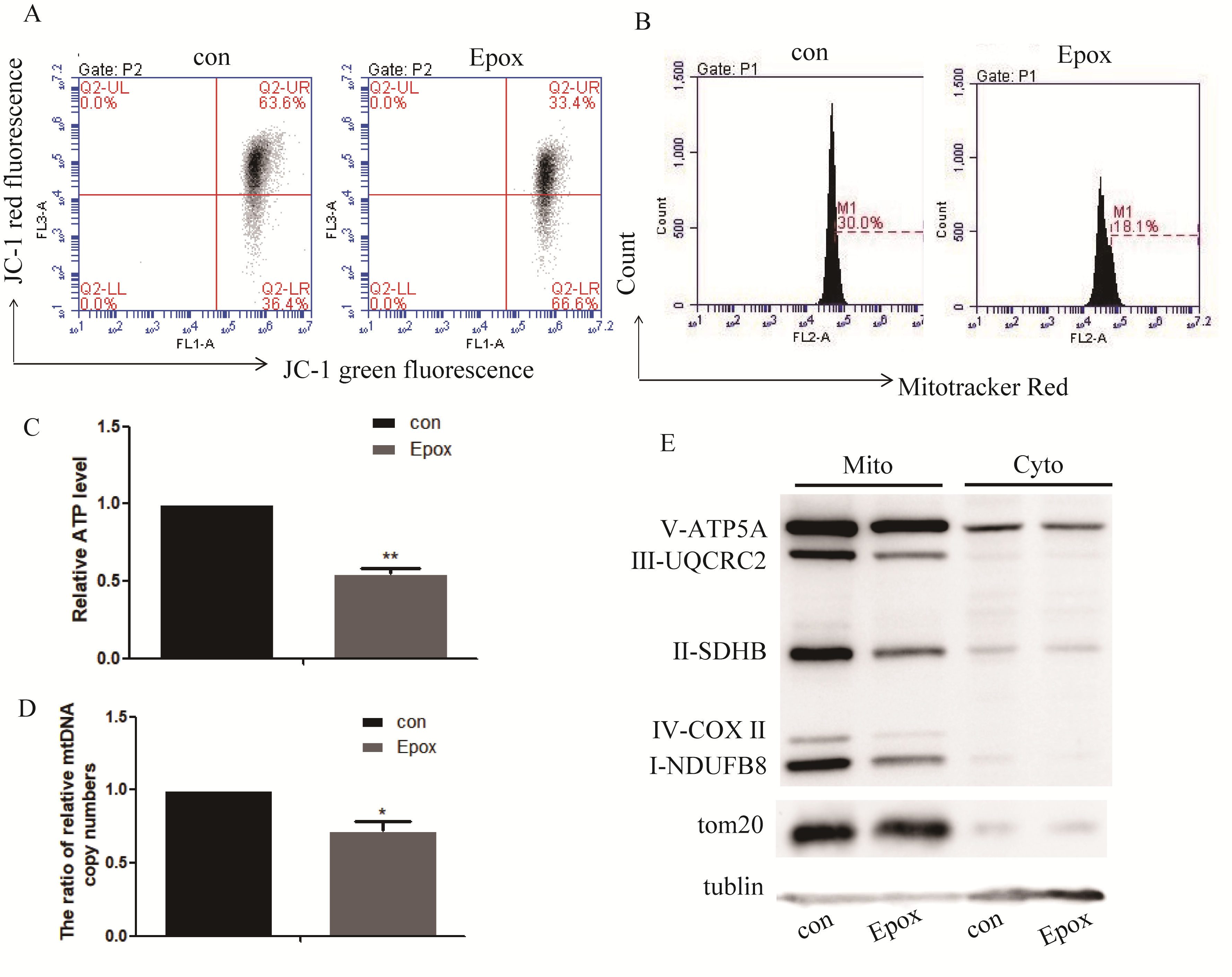

Supplement: Supplementary Materials — EPOX decreases mitochobdrial function in A2780 cells. [file 4830418.f1.doc]
